# Baricitinib versus tocilizumab in mechanically ventilated patients with COVID-19: a nationwide cohort study

**DOI:** 10.1186/s13054-024-05063-2

**Published:** 2024-08-29

**Authors:** Seung-Hun You, Moon Seong Baek, Tae Wan Kim, Sun-Young Jung, Won-Young Kim

**Affiliations:** 1 Department of Global Innovative Drugs, The Graduate School of Chung-Ang University, Chung-Ang University, Seoul, Republic of Korea; 2 Division of Pulmonary and Critical Care Medicine, Department of Internal Medicine, Chung-Ang University Hospital, Chung-Ang University College of Medicine, Seoul, Republic of Korea; 3 College of Pharmacy, Chung-Ang University, Seoul, Republic of Korea

**Dear Editor,**

No large-scale study has compared baricitinib with tocilizumab specifically for critical coronavirus disease 2019 (COVID-19). An exploratory trial that included patients with COVID-19 on mechanical ventilation (MV) or extracorporeal membrane oxygenation (ECMO) demonstrated a marked reduction in 28-day mortality in the baricitinib group, although baricitinib was compared to placebo [1]. Most of the studies that have conducted head-to-head comparisons between baricitinib and tocilizumab in patients with severe COVID-19 had lower rates of disease severity (< 5% on MV) [2]. Thus, which of the two drugs is more beneficial for patients with rapidly progressing inflammatory response is unclear. Additionally, most patients in previous studies were unvaccinated, thus limiting the stratified analysis according to vaccination status.

To address the current knowledge gaps, this study was performed as a large-scale analysis of Korean health insurance claims data to compare the efficacies of baricitinib versus tocilizumab in patients with COVID-19 receiving MV.

Adult patients (age ≥ 18 years) with confirmed COVID-19 admitted from October 8, 2020 to October 31, 2022, who required MV, were analyzed. Patients who received at least one dose of baricitinib or tocilizumab during the index hospitalization were assessed. The exclusion criteria were age < 18 years, death or discharge within the first 2 days of hospitalization, cardiac arrest, palliative care, pregnancy or related conditions, and co-administration of baricitinib and tocilizumab. Propensity score (PS) matching was conducted to control for differences in the baseline variables of patients receiving either baricitinib or tocilizumab. For the PS model, baricitinib use was employed as the dependent variable, and the independent variables were all the baseline covariates listed in Table S1. Covariate balance before and after matching was evaluated by standardized mean differences, and a difference of < 0.10 was considered well-balanced. Logistic regression analyses were performed to compute the odds ratios (ORs) and 95% confidence intervals (CIs) of the outcomes associated with baricitinib use. Subgroup analyses for the outcomes were performed according to age, sex, Charlson Comorbidity Index, neuromuscular blocking agents, renal replacement therapy, and ECMO. To determine the possible confounding by the COVID-19 vaccination, baseline and outcome analyses were stratified according to the vaccination status prior to admission. All statistical analyses were performed using SAS software (version 9.4; SAS Institute, Cary, NC, USA).

Among 1630 included patients (mean [standard deviation] age, 71.4 [12.8] years; men, 58.6%), PS matching resulted in 557 patients in each group (Fig. S1). No significant differences were observed in the baseline characteristics between the PS-matched groups (Table S1). For the unmatched and PS-matched groups, the median (interquartile range) durations of baricitinib use and tocilizumab use were 8 (4–13) days and 1 (1–1) day, respectively. On day 30, significantly fewer patients died in the baricitinib group (49.4% vs. 57.8%; OR 0.71; 95% CI 0.56–0.90; Fig. 1a). This corroborates the study by Ely et al. [1], although the mortality rate in the baricitinib group (39%) was lower than that of current study. Baricitinib exhibits a more favorable response to tocilizumab possibly because of its different mechanisms of action. Although tocilizumab specifically inhibits a single cytokine [3], baricitinib inhibits multiple inflammatory pathways [4]. The administration of baricitinib over multiple days may result in the delivery of more consistent drug concentrations that maintain its anti-inflammatory effect [4].

**Fig. 1:**
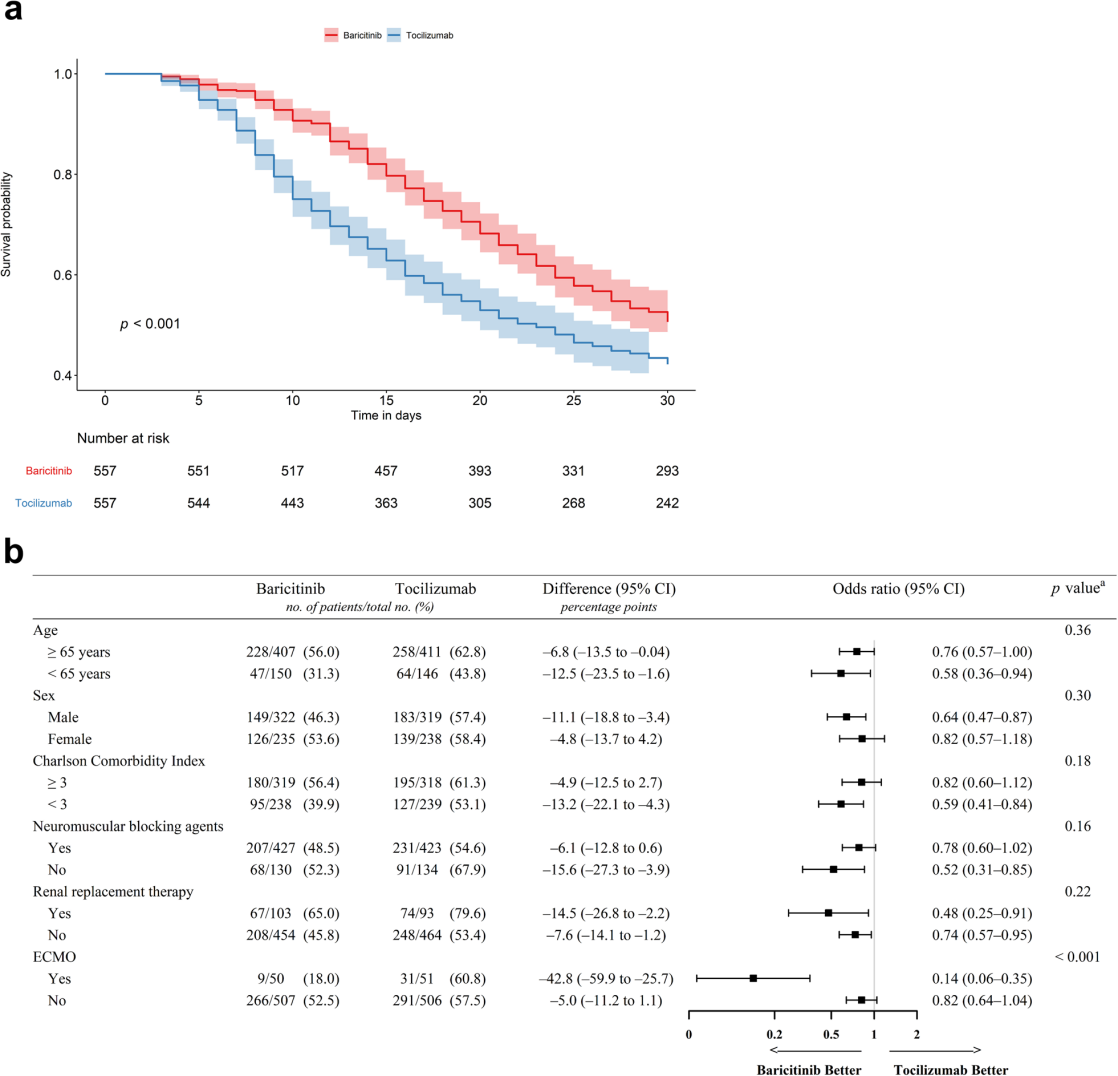
**a** Kaplan–Meier curves of the probability of survival within 30 days. **b** Association of baricitinib on 30-day mortality by subgroups. Odds ratios (represented by squares) and 95% CIs (corresponding lines through them) are calculated for the propensity score-matched baricitinib (n = 557) and tocilizumab (n = 557) groups. ^a^*p* values are for the interaction term. *CI* confidence interval, *ECMO* extracorporeal membrane oxygenation

The association between baricitinib and 30-day mortality was consistent across all subgroups (Fig. 1b). Although limited by the small number of patients, the mortality risk was the lowest among patients receiving baricitinib and ECMO (18.0% vs. 60.8%; OR 0.14; 95% CI 0.06–0.35; *p* < 0.001 for interaction). This suggests that the beneficial effects of baricitinib may be more evident in patients who exhibit an increased inflammatory response. The baseline characteristics of the cohort that included only unvaccinated patients are shown in Table S2. Most baseline characteristics of the PS-matched baricitinib and tocilizumab groups were similar, except for age. After adjustment for age, patients administered baricitinib exhibited significantly lower 30-day mortality rates (46.8% vs. 59.7%; OR 0.61; 95% CI 0.44–0.87) than those who received tocilizumab. This finding is consistent with that of the study by Trøseid et al. [5] of unvaccinated participants that demonstrated lower 60-day mortality in the baricitinib group than in the placebo group. However, the unvaccinated patients were younger and had less comorbidities in both studies. The baseline characteristics of the cohort that included only vaccinated patients are shown in Table S3. Vaccination itself may not significantly affect the treatment response to baricitinib or tocilizumab once severe COVID-19 has developed. Among vaccinated patients, no significant difference was detected between the baricitinib and tocilizumab groups in the 30-day mortality (49.6% vs. 55.3%; OR 0.80; 95% CI 0.56–1.13).

For patients with COVID-19 requiring MV, baricitinib was associated with lower 30-day mortality than tocilizumab. Additional studies are needed to evaluate the efficacy of baricitinib in specific subgroups of patients with critical COVID-19.

## Supplementary Information

Additional file1

## Data Availability

All data generated or analyzed during this study are included in this published article and its supplementary information files.

## Abbreviations

CI: Confidence interval
COVID-19: Coronavirus disease 2019
ECMO: Extracorporeal membrane oxygenation
MV: Mechanical ventilation
OR: Odds ratio
PS: Propensity score
