# Supplementary material for: Baricitinib versus tocilizumab in mechanically ventilated patients with COVID-19: a nationwide cohort study
Source: Crit Care. 2024 Aug 29;28:282. doi: 10.1186/s13054-024-05063-2 (PMC11363553; doi:10.1186/s13054-024-05063-2)
Supplement: Supplementary file 1 — Additional file1 [file 13054_2024_5063_MOESM1_ESM.docx]

**Table S1** Baseline characteristics of patients in the baricitinib and tocilizumab groups

| Characteristics | Unmatched cohort | | | Matched cohort | | |
| --- | --- | --- | --- | --- | --- | --- |
|  | Baricitinib  (n = 619) | Tocilizumab  (n = 1011) | SMD | Baricitinib  (n = 557) | Tocilizumab  (n = 557) | SMD |
| Age, mean (SD), y | 69.8 (13.2) | 72.4 (12.5) | –0.20 | 70.1 (13.0) | 70.6 (13.3) | –0.04 |
| Sex, No. (%) |  |  | –0.05 |  |  | 0.01 |
| Male | 353 (57.0) | 602 (59.5) |  | 322 (57.8) | 319 (57.3) |  |
| Female | 266 (43.0) | 409 (40.5) |  | 235 (42.2) | 238 (42.7) |  |
| Comorbidities, No. (%) |  |  |  |  |  |  |
| Diabetes | 282 (45.6) | 512 (50.6) | –0.10 | 258 (46.3) | 252 (45.2) | 0.02 |
| Hypertension | 405 (65.4) | 712 (70.4) | –0.11 | 368 (66.1) | 364 (65.4) | 0.02 |
| Myocardial infarction | 29 (4.7) | 50 (4.9) | –0.01 | 27 (4.8) | 28 (5.0) | –0.008 |
| Congestive heart failure | 92 (14.9) | 210 (20.8) | –0.15 | 89 (16.0) | 86 (15.4) | 0.01 |
| Cerebrovascular disease | 146 (23.6) | 246 (24.3) | –0.02 | 128 (23.0) | 133 (23.9) | –0.02 |
| Chronic pulmonary disease | 237 (38.3) | 497 (49.2) | –0.22 | 224 (40.2) | 224 (40.2) | < 0.001 |
| Chronic liver disease | 184 (29.7) | 303 (30.0) | –0.005 | 170 (30.5) | 177 (31.8) | –0.03 |
| Chronic kidney disease | 77 (12.4) | 209 (20.7) | –0.22 | 70 (12.6) | 61 (11.0) | 0.05 |
| Malignancy | 87 (14.1) | 192 (19.0) | –0.13 | 85 (15.3) | 80 (14.4) | 0.03 |
| Charlson Comorbidity Index, mean (SD) | 3.4 (2.8) | 4.2 (3.1) | –0.28 | 3.5 (2.9) | 3.6 (2.9) | –0.04 |
| Immunosuppression, No. (%) | 107 (17.3) | 254 (25.1) | –0.19 | 102 (18.3) | 99 (17.8) | 0.01 |
| Income level, No. (%) |  |  | 0.09 |  |  | 0.02 |
| Q1 (lowest) | 189 (30.5) | 297 (29.4) |  | 168 (30.2) | 170 (30.5) |  |
| Q2 | 101 (16.3) | 145 (14.3) |  | 91 (16.3) | 89 (16.0) |  |
| Q3 | 121 (19.5) | 207 (20.5) |  | 107 (19.2) | 108 (19.4) |  |
| Q4 (highest) | 208 (33.6) | 362 (35.8) |  | 191 (34.3) | 190 (34.1) |  |
| Hospital size, No. (%) |  |  | 0.36 |  |  | 0.04 |
| < 500 beds | 316 (51.1) | 365 (36.1) |  | 265 (47.6) | 259 (46.5) |  |
| 500–1000 beds | 205 (33.1) | 510 (50.4) |  | 199 (35.7) | 209 (37.5) |  |
| ≥ 1000 beds | 98 (15.8) | 136 (13.5) |  | 93 (16.7) | 89 (16.0) |  |
| COVID-19 vaccination, No. (%) | 271 (43.8) | 555 (54.9) | –0.22 | 255 (45.8) | 253 (45.4) | 0.007 |
| Organ dysfunction, No. (%) |  |  |  |  |  |  |
| Cardiovascular | 567 (91.6) | 951 (94.1) | –0.10 | 513 (92.1) | 512 (91.9) | 0.007 |
| Respiratory | 619 (100) | 1011 (100) | < 0.001 | 557 (100) | 557 (100) | < 0.001 |
| Neurologic | 68 (11.0) | 91 (9.0) | 0.07 | 56 (10.1) | 56 (10.1) | < 0.001 |
| Hematologic | 74 (12.0) | 147 (14.5) | –0.08 | 68 (12.2) | 65 (11.7) | 0.02 |
| Hepatic | 12 (1.9) | 18 (1.8) | 0.01 | 11 (2.0) | 11 (2.0) | < 0.001 |
| Renal | 176 (28.4) | 414 (40.9) | –0.27 | 168 (30.2) | 157 (28.2) | 0.04 |
| Metabolic | 28 (4.5) | 51 (5.0) | –0.02 | 26 (4.7) | 27 (4.8) | –0.008 |
| Corticosteroids, No. (%) | 597 (96.4) | 1007 (99.6) | –0.23 | 553 (99.3) | 553 (99.3) | < 0.001 |
| Neuromuscular blocking agents, No. (%) | 482 (77.9) | 717 (70.9) | 0.16 | 427 (76.7) | 423 (75.9) | 0.02 |
| Vasopressor use, No. (%) | 564 (91.1) | 946 (93.6) | –0.09 | 511 (91.7) | 508 (91.2) | 0.02 |
| Renal replacement therapy, No. (%) | 107 (17.3) | 286 (28.3) | –0.26 | 103 (18.5) | 93 (16.7) | 0.05 |
| ECMO, No. (%) | 58 (9.4) | 66 (6.5) | 0.11 | 50 (9.0) | 51 (9.2) | –0.006 |

*COVID-19* coronavirus disease 2019, *ECMO* extracorporeal membrane oxygenation, *SD* standard deviation, *SMD* standardized mean difference

**Table S2** Baseline characteristics of the patients in the baricitinib and tocilizumab groups among those who were not vaccinated against COVID-19

| Characteristics | Unmatched cohort | | | Matched cohort | | |
| --- | --- | --- | --- | --- | --- | --- |
|  | Baricitinib  (n = 348) | Tocilizumab  (n = 456) | SMD | Baricitinib  (n = 295) | Tocilizumab  (n = 295) | SMD |
| Age, mean (SD), y | 67.6 (14.2) | 71.3 (14.0) | –0.26 | 68.0 (13.7) | 69.7 (14.8) | –0.12 |
| Sex, No. (%) |  |  | –0.05 |  |  | –0.04 |
| Male | 186 (53.4) | 254 (55.7) |  | 161 (54.6) | 167 (56.6) |  |
| Female | 162 (46.6) | 202 (44.3) |  | 134 (45.4) | 128 (43.4) |  |
| Comorbidities, No. (%) |  |  |  |  |  |  |
| Diabetes | 137 (39.4) | 211 (46.3) | –0.14 | 120 (40.7) | 123 (41.7) | –0.02 |
| Hypertension | 197 (56.6) | 308 (67.5) | –0.23 | 177 (60.0) | 178 (60.3) | –0.007 |
| Myocardial infarction | 16 (4.6) | 24 (5.3) | –0.03 | 14 (4.7) | 11 (3.7) | 0.05 |
| Congestive heart failure | 45 (12.9) | 87 (19.1) | –0.17 | 42 (14.2) | 46 (15.6) | –0.04 |
| Cerebrovascular disease | 69 (19.8) | 114 (25.0) | –0.12 | 63 (21.4) | 60 (20.3) | 0.03 |
| Chronic pulmonary disease | 119 (34.2) | 205 (45.0) | –0.22 | 109 (36.9) | 107 (36.3) | 0.01 |
| Chronic liver disease | 86 (24.7) | 126 (27.6) | –0.07 | 70 (23.7) | 78 (26.4) | –0.06 |
| Chronic kidney disease | 39 (11.2) | 80 (17.5) | –0.18 | 36 (12.2) | 29 (9.8) | 0.08 |
| Malignancy | 45 (12.9) | 71 (15.6) | –0.08 | 39 (13.2) | 44 (14.9) | –0.05 |
| Charlson Comorbidity Index, mean (SD) | 2.9 (2.8) | 3.8 (3.1) | –0.29 | 3.0 (2.8) | 3.1 (2.8) | –0.02 |
| Immunosuppression, No. (%) | 49 (14.1) | 80 (17.5) | –0.10 | 43 (14.6) | 47 (15.9) | –0.04 |
| Income level, No. (%) |  |  | 0.14 |  |  | 0.03 |
| Q1 (lowest) | 114 (32.8) | 129 (28.3) |  | 95 (32.2) | 91 (30.8) |  |
| Q2 | 54 (15.5) | 67 (14.7) |  | 45 (15.3) | 45 (15.3) |  |
| Q3 | 71 (20.4) | 93 (20.4) |  | 59 (20.0) | 61 (20.7) |  |
| Q4 (highest) | 109 (31.3) | 167 (36.6) |  | 96 (32.5) | 98 (33.2) |  |
| Hospital size, No. (%) |  |  | 0.36 |  |  | < 0.001 |
| < 500 beds | 181 (52.0) | 161 (35.3) |  | 137 (46.4) | 137 (46.4) |  |
| 500–1000 beds | 118 (33.9) | 223 (48.9) |  | 112 (38.0) | 111 (37.6) |  |
| ≥ 1000 beds | 49 (14.1) | 72 (15.8) |  | 46 (15.6) | 47 (15.9) |  |
| Organ dysfunction, No. (%) |  |  |  |  |  |  |
| Cardiovascular | 317 (91.1) | 440 (96.5) | –0.23 | 279 (94.6) | 280 (94.9) | –0.02 |
| Respiratory | 348 (100) | 456 (100) | < 0.001 | 295 (100) | 295 (100) | < 0.001 |
| Neurologic | 33 (9.5) | 40 (8.8) | 0.02 | 27 (9.2) | 23 (7.8) | 0.05 |
| Hematologic | 44 (12.6) | 66 (14.5) | –0.05 | 39 (13.2) | 38 (12.9) | 0.01 |
| Hepatic | 8 (2.3) | 7 (1.5) | 0.06 | 6 (2.0) | 6 (2.0) | < 0.001 |
| Renal | 96 (27.6) | 184 (40.4) | –0.27 | 92 (31.2) | 83 (28.1) | 0.07 |
| Metabolic | 21 (6.0) | 27 (5.9) | 0.005 | 17 (5.8) | 18 (6.1) | –0.01 |
| Corticosteroids, No. (%) | 339 (97.4) | 455 (99.8) | –0.20 | 295 (100) | 294 (99.7) | 0.08 |
| Neuromuscular blocking agents, No. (%) | 274 (78.7) | 332 (72.8) | 0.14 | 229 (77.6) | 227 (76.9) | 0.02 |
| Vasopressor use, No. (%) | 317 (91.1) | 437 (95.8) | –0.19 | 279 (94.6) | 280 (94.9) | –0.02 |
| Renal replacement therapy, No. (%) | 58 (16.7) | 133 (29.2) | –0.30 | 56 (19.0) | 52 (17.6) | 0.04 |
| ECMO, No. (%) | 40 (11.5) | 37 (8.1) | 0.11 | 31 (10.5) | 32 (10.8) | –0.01 |

*COVID-19* coronavirus disease 2019, *ECMO* extracorporeal membrane oxygenation, *SD* standard deviation, *SMD* standardized mean difference

**Table S3** Baseline characteristics of patients in the baricitinib and tocilizumab groups among those who were vaccinated against COVID-19

| Characteristics | Unmatched cohort | | | Matched cohort | | |
| --- | --- | --- | --- | --- | --- | --- |
|  | Baricitinib  (n = 271) | Tocilizumab  (n = 555) | SMD | Baricitinib  (n = 246) | Tocilizumab  (n = 246) | SMD |
| Age, mean (SD), y | 72.7 (11.1) | 73.4 (11.0) | –0.06 | 72.7 (11.1) | 72.2 (12.0) | 0.04 |
| Sex, No. (%) |  |  | –0.02 |  |  | –0.03 |
| Male | 167 (61.6) | 348 (62.7) |  | 150 (61.0) | 153 (62.2) |  |
| Female | 104 (38.4) | 207 (37.3) |  | 96 (39.0) | 93 (37.8) |  |
| Comorbidities, No. (%) |  |  |  |  |  |  |
| Diabetes | 145 (53.5) | 301 (54.2) | –0.01 | 131 (53.3) | 130 (52.8) | 0.008 |
| Hypertension | 208 (76.8) | 404 (72.8) | 0.09 | 186 (75.6) | 195 (79.3) | –0.09 |
| Myocardial infarction | 13 (4.8) | 26 (4.7) | 0.005 | 12 (4.9) | 10 (4.1) | 0.04 |
| Congestive heart failure | 47 (17.3) | 123 (22.2) | –0.12 | 44 (17.9) | 38 (15.4) | 0.07 |
| Cerebrovascular disease | 77 (28.4) | 132 (23.8) | 0.11 | 66 (26.8) | 71 (28.9) | –0.05 |
| Chronic pulmonary disease | 118 (43.5) | 292 (52.6) | –0.18 | 108 (43.9) | 107 (43.5) | 0.008 |
| Chronic liver disease | 98 (36.2) | 177 (31.9) | 0.09 | 88 (35.8) | 80 (32.5) | 0.07 |
| Chronic kidney disease | 38 (14.0) | 129 (23.2) | –0.24 | 34 (13.8) | 35 (14.2) | –0.01 |
| Malignancy | 42 (15.5) | 121 (21.8) | –0.16 | 40 (16.3) | 42 (17.1) | –0.02 |
| Charlson Comorbidity Index, mean (SD) | 4.0 (2.8) | 4.6 (3.0) | –0.21 | 3.9 (2.8) | 4.0 (2.6) | –0.02 |
| Immunosuppression, No. (%) | 58 (21.4) | 174 (31.4) | –0.23 | 54 (22.0) | 58 (23.6) | –0.04 |
| Income level, No. (%) |  |  | 0.11 |  |  | 0.03 |
| Q1 (lowest) | 75 (27.7) | 168 (30.3) |  | 68 (27.6) | 70 (28.5) |  |
| Q2 | 47 (17.3) | 78 (14.1) |  | 41 (17.6) | 41 (16.7) |  |
| Q3 | 50 (18.5) | 114 (20.5) |  | 44 (17.9) | 42 (17.1) |  |
| Q4 (highest) | 99 (36.5) | 195 (35.1) |  | 93 (37.8) | 93 (37.8) |  |
| Hospital size, No. (%) |  |  | 0.42 |  |  | 0.06 |
| < 500 beds | 135 (49.8) | 204 (36.8) |  | 118 (48.0) | 117 (47.6) |  |
| 500–1000 beds | 87 (32.1) | 287 (51.7) |  | 84 (34.1) | 88 (35.8) |  |
| ≥ 1000 beds | 49 (18.1) | 64 (11.5) |  | 44 (17.9) | 41 (16.7) |  |
| Organ dysfunction, No. (%) |  |  |  |  |  |  |
| Cardiovascular | 250 (92.3) | 511 (92.1) | 0.007 | 227 (92.3) | 225 (91.5) | 0.03 |
| Respiratory | 271 (100) | 555 (100) | < 0.001 | 246 (100) | 246 (100) | < 0.001 |
| Neurologic | 35 (12.9) | 51 (9.2) | 0.12 | 27 (11.0) | 32 (13.0) | –0.06 |
| Hematologic | 30 (11.1) | 81 (14.6) | –0.11 | 27 (11.0) | 28 (11.4) | –0.01 |
| Hepatic | 4 (1.5) | 11 (2.0) | –0.04 | 4 (1.6) | 5 (2.0) | –0.03 |
| Renal | 80 (29.5) | 230 (41.4) | –0.25 | 75 (30.5) | 82 (33.3) | –0.06 |
| Metabolic | 7 (2.6) | 24 (4.3) | –0.10 | 6 (2.4) | 9 (3.7) | –0.07 |
| Corticosteroids, No. (%) | 258 (95.2) | 552 (99.5) | –0.27 | 244 (99.2) | 243 (98.8) | 0.04 |
| Neuromuscular blocking agents, No. (%) | 208 (76.8) | 385 (69.4) | 0.17 | 188 (76.4) | 186 (75.6) | 0.02 |
| Vasopressor use, No. (%) | 247 (91.1) | 509 (91.7) | –0.02 | 225 (91.5) | 224 (91.1) | 0.01 |
| Renal replacement therapy, No. (%) | 49 (18.1) | 153 (27.6) | –0.23 | 46 (18.7) | 48 (19.5) | –0.02 |
| ECMO, No. (%) | 18 (6.6) | 29 (5.2) | 0.06 | 17 (6.9) | 17 (6.9) | < 0.001 |

*COVID-19* coronavirus disease 2019, *ECMO* extracorporeal membrane oxygenation, *SD* standard deviation, *SMD* standardized mean difference

**Fig. S1** Study flowchart


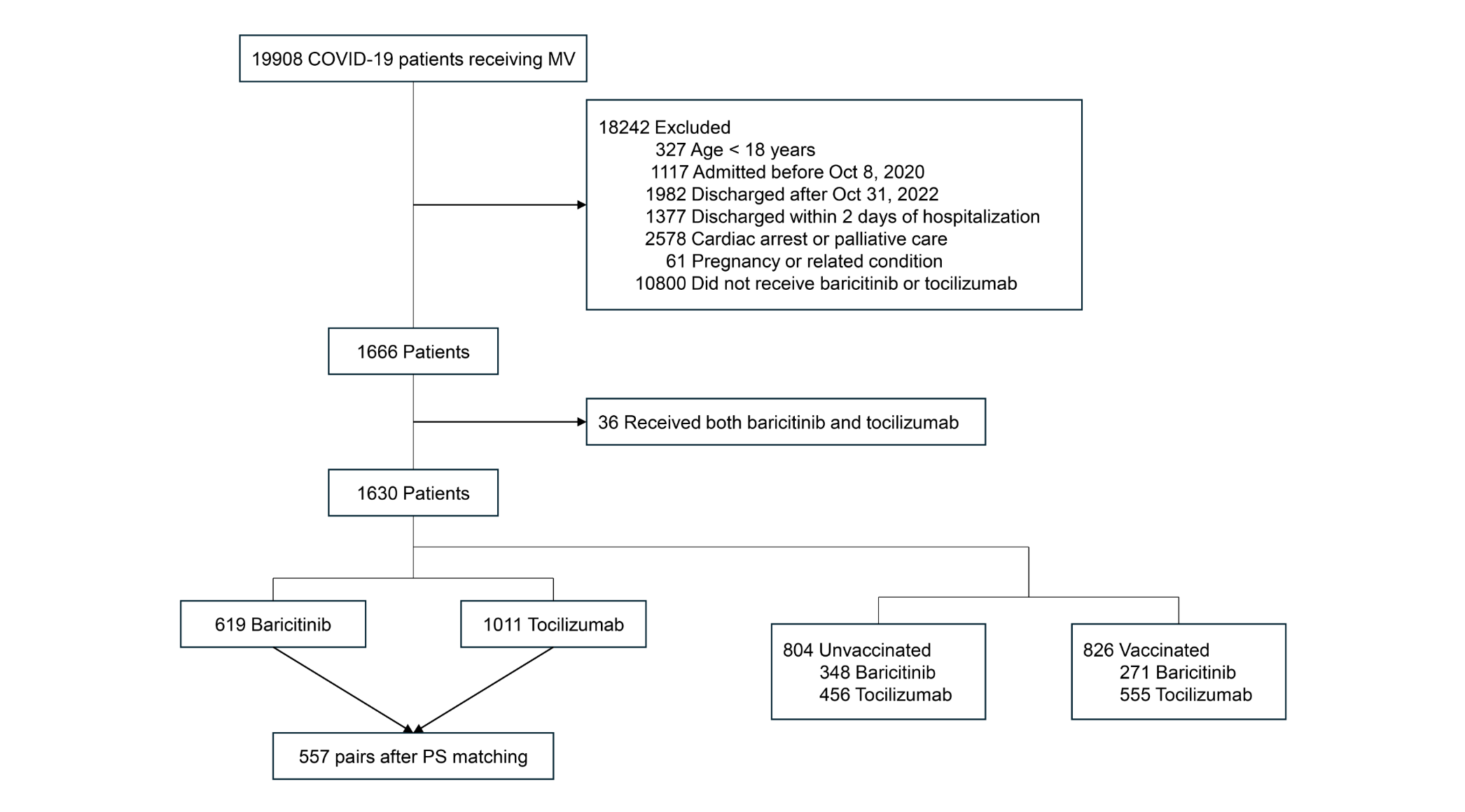


*COVID-19* coronavirus disease 2019, *MV* mechanical ventilation, *PS* propensity score
